# Supplementary material for: Interleukin-6 from Mycobacterium abscessus-infected macrophages enhances the survival of B cell-derived plasmablasts in vitro
Source: Microbiol Spectr. 2026 Apr 20;14(6):e02520-25. doi: 10.1128/spectrum.02520-25 (PMC13228045; doi:10.1128/spectrum.02520-25)
Supplement: Data S1 to S3 — Monocyte and macrophage surface marker expression, macrophage cell death and cytokine gene expression after Mab infection, and IL-6 levels under different infection conditions. [file spectrum.02520-25-s0001.pdf]

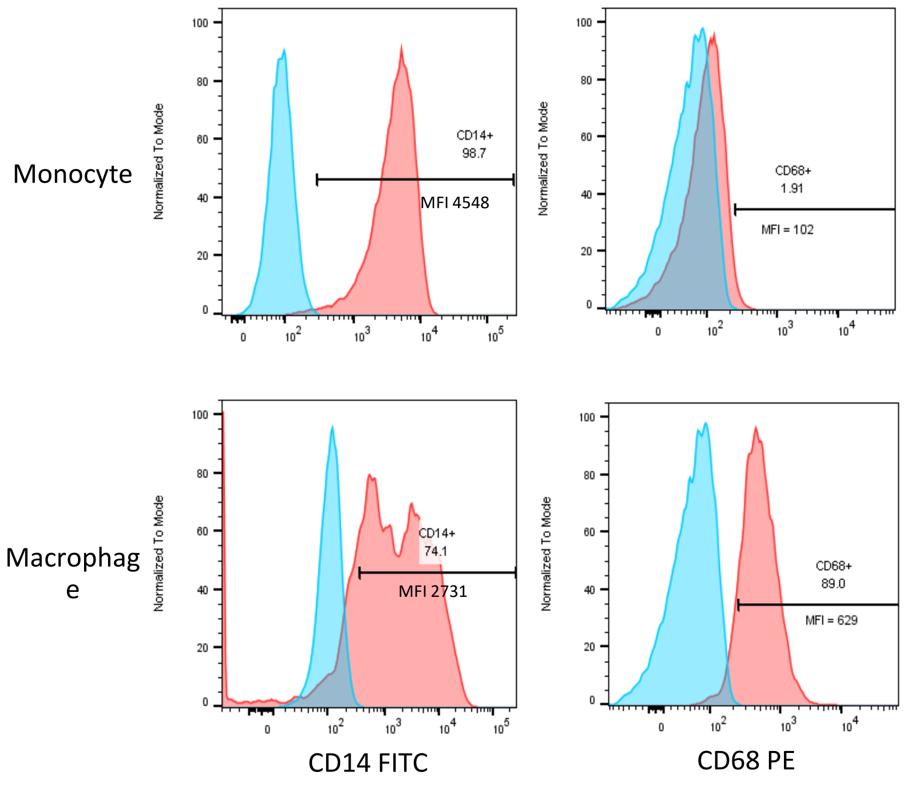

Supplementary Data 1: Monocyte and macrophage surface marker expression

Monocytes (day 0) and macrophages (day 7) were detached using PBS containing 2 mM EDTA, stained with FITC-conjugated anti-CD14 (clone HCD14) and PE-conjugated anti-CD68 (clone Y1/82A, BioLegend), and analyzed by flow cytometry. Histograms show isotype control (blue) and specific antibody staining (red).

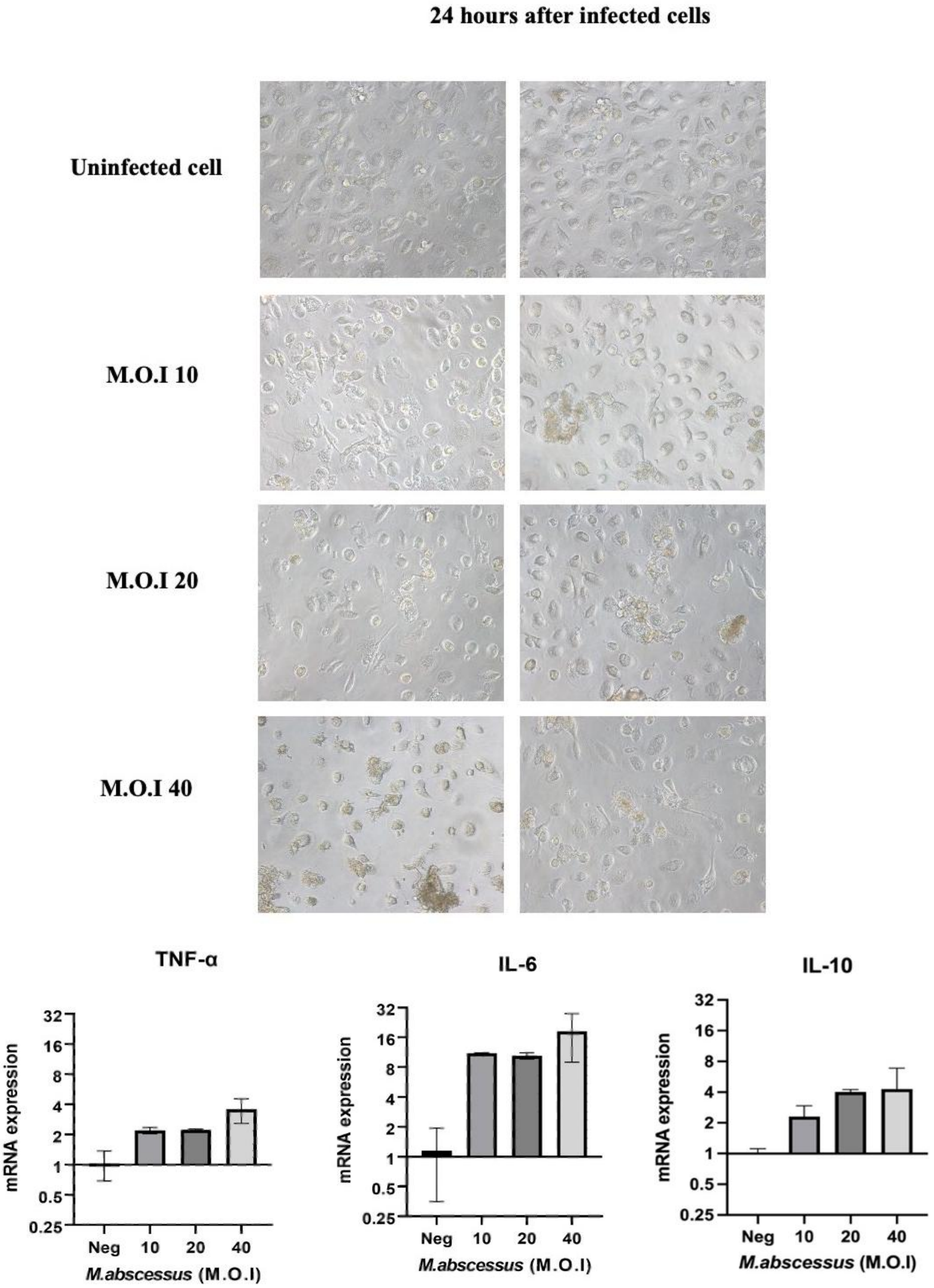

Supplementary Data 2: Macrophage cell death at 24 h and cytokine gene expression at 6 h after infection with Mab at different MOIs.

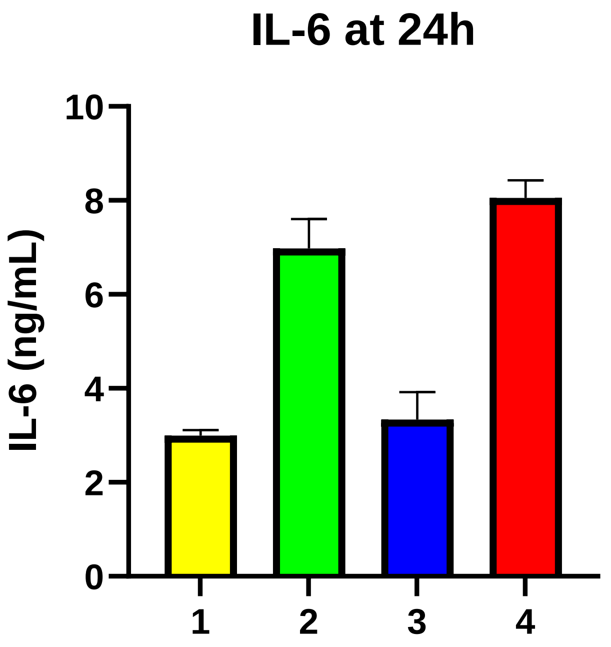

Supplementary Data 3: IL-6 levels under different infection conditions.

Macrophages were infected with *M. abscessus* at a multiplicity of infection (MOI) of 20:1 using the following conditions:

1. Inoculum removed after 2 hours; cells maintained in medium containing amikacin (20 mg/L)
2. Inoculum removed after 2 hours; cells maintained in medium without antibiotics
3. Inoculum not removed; cells maintained in medium containing amikacin (20 mg/L)
4. Standard protocol: inoculum not removed and no antibiotics

Data are presented as mean ± SD from three independent experiments
