# Supplementary material for: Interleukin-6 from Mycobacterium abscessus-infected macrophages enhances the survival of B cell-derived plasmablasts in vitro
Source: Microbiol Spectr. 2026 Apr 20;14(6):e02520-25. doi: 10.1128/spectrum.02520-25 (PMC13228045; doi:10.1128/spectrum.02520-25)

Supplementary Data 4

Flow cytometry data showing:

- B cell purity on day 0
- Plasmablasts on day 4 with increased frequency of T cells
- Plasmablasts on day 7 with variable CD138 expression

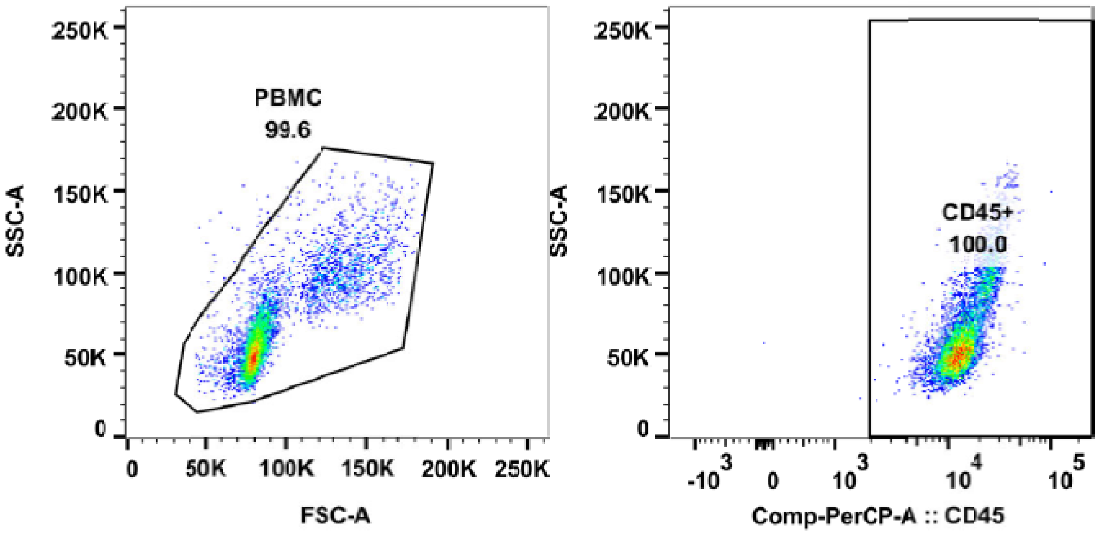

CD45+ leukocyte
No RBC or cell debris

B cell purity

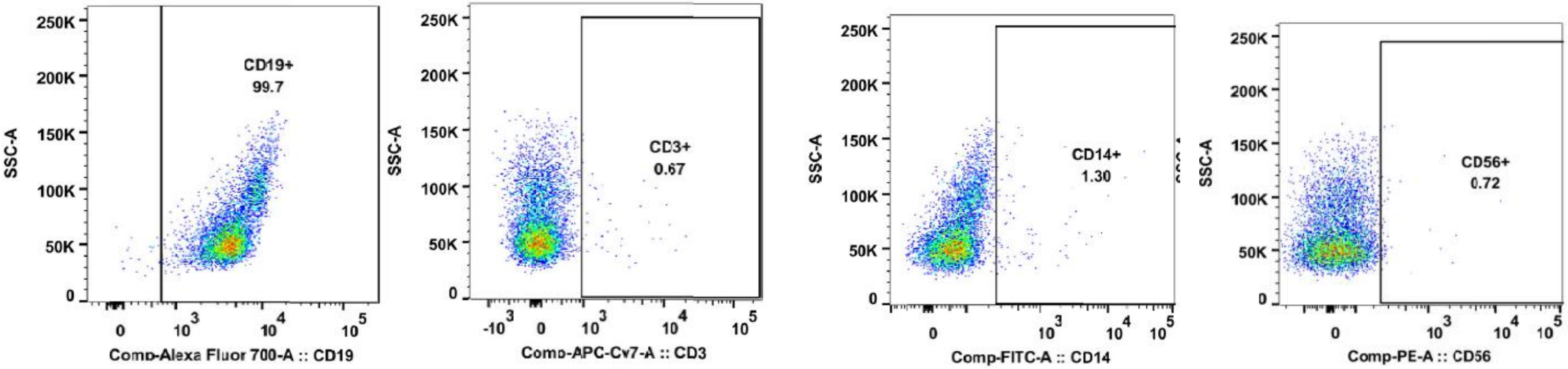

Day 0

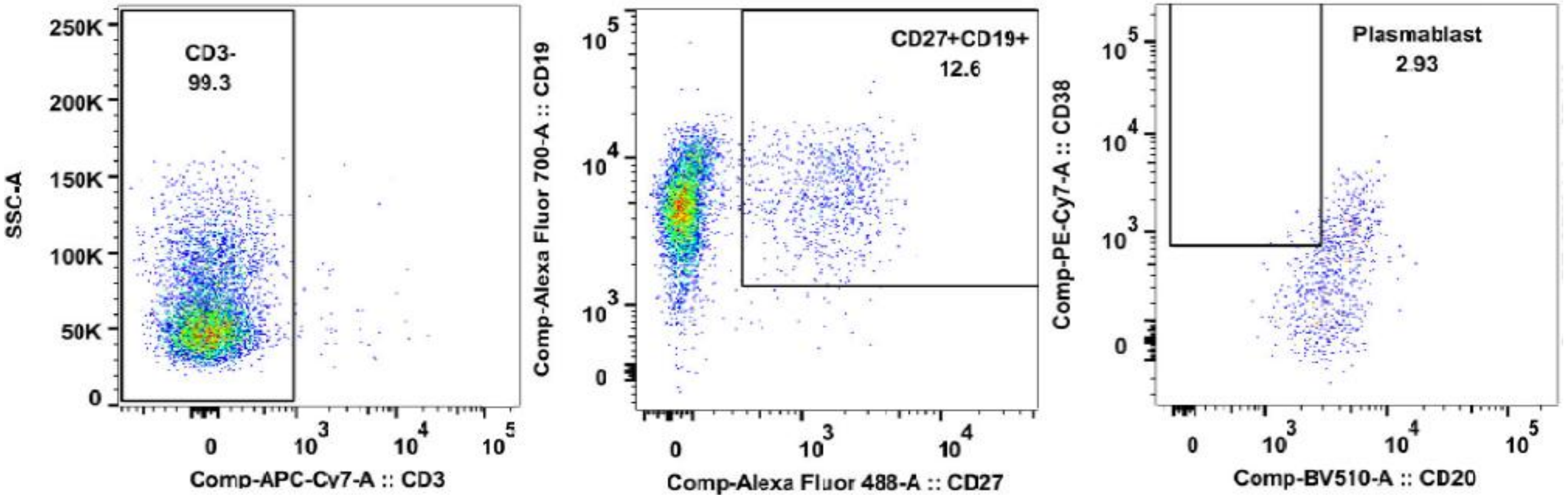
CD3-
99.3
SSC-A
250K
200K
150K
100K
50K
0
Comp-APC-Cy7-A :: CD3
CD27+CD19+
12.6
Comp-Alexa Fluor 700-A :: CD19
Comp-Alexa Fluor 488-A :: CD27
Plasmablast
2.93
Comp-PE-Cy7-A :: CD38
Comp-BV510-A :: CD20

Day 4

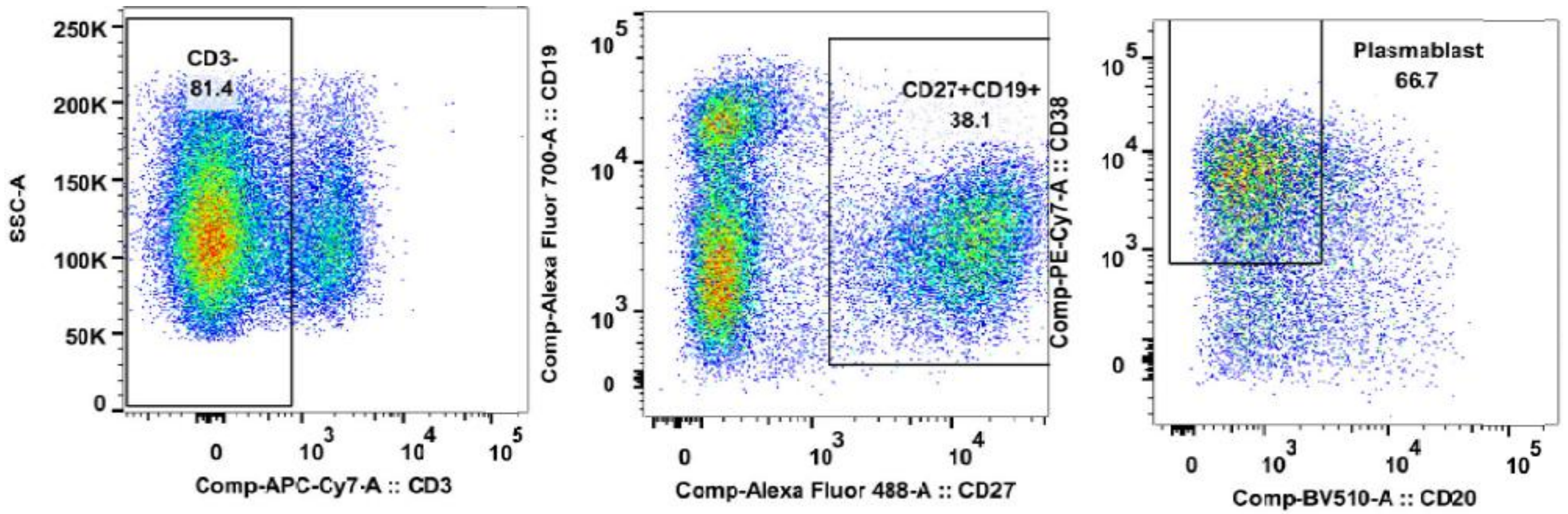
CD3-
81.4
SSC-A
250K
200K
150K
100K
50K
0
Comp-APC-Cy7-A :: CD3
CD27+CD19+
38.1
Comp-Alexa Fluor 700-A :: CD19
Comp-Alexa Fluor 488-A :: CD27
Plasmablast
66.7
Comp-PE-Cy7-A :: CD38
Comp-BV510-A :: CD20

Day 7 (condition with IL-6, after T cell and NK cell depletion on day 4)

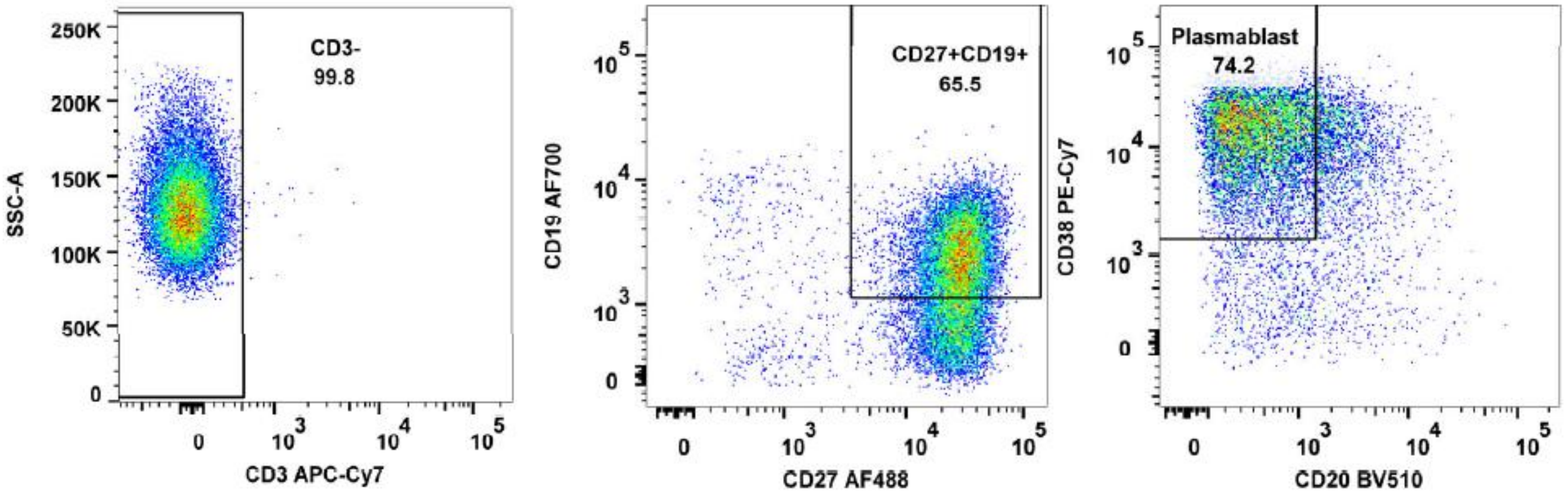

Gated from CD27^{hi},CD38^{hi}, CD20^{lo}

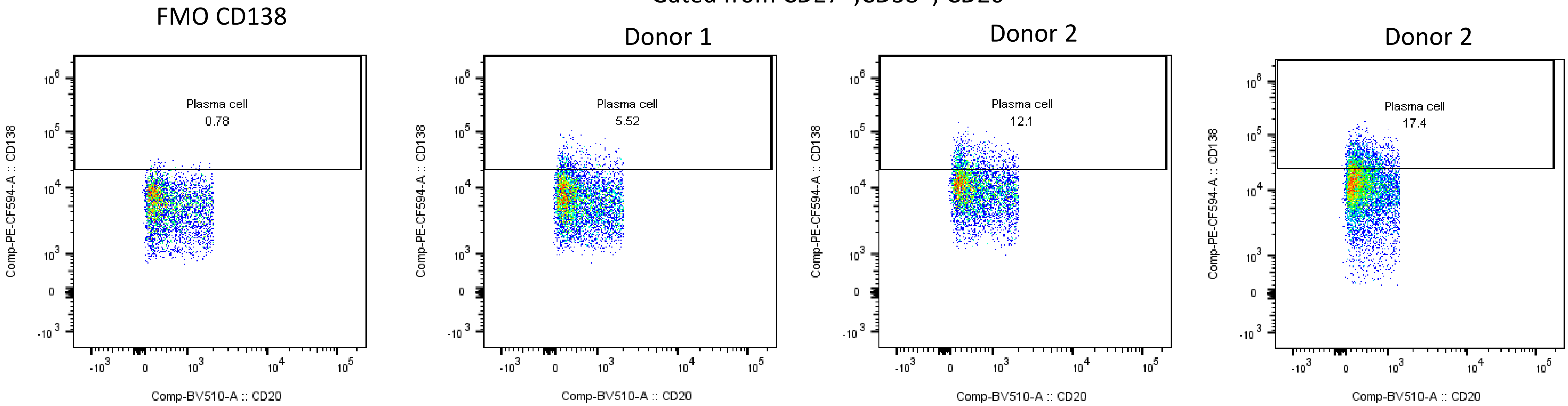

Supplement: Data S4 — Flow cytometry data. [file spectrum.02520-25-s0002.pdf]
