# Supplementary figures and images for: Interleukin-6 from Mycobacterium abscessus-infected macrophages enhances the survival of B cell-derived plasmablasts in vitro
Source: Microbiol Spectr. 2026 Apr 20;14(6):e02520-25. doi: 10.1128/spectrum.02520-25 (PMC13228045; doi:10.1128/spectrum.02520-25)

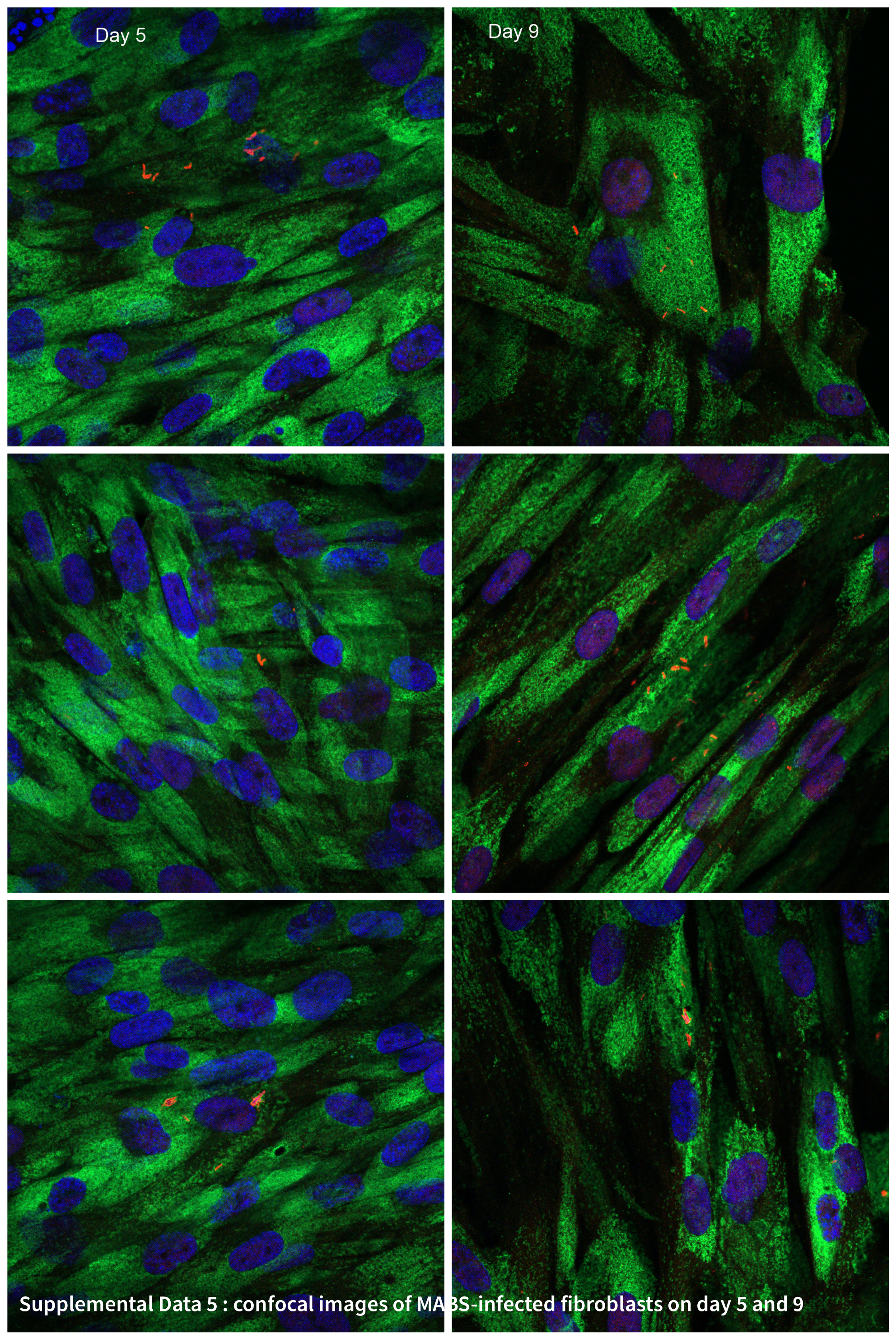

Supplement: Data S5 — Confocal microscopy images. [file spectrum.02520-25-s0003.tiff]
