# Supplementary material for: Interleukin-6 from Mycobacterium abscessus-infected macrophages enhances the survival of B cell-derived plasmablasts in vitro
Source: Microbiol Spectr. 2026 Apr 20;14(6):e02520-25. doi: 10.1128/spectrum.02520-25 (PMC13228045; doi:10.1128/spectrum.02520-25)
Supplement: Data S6 and S7 — Plasmablasts on day 7 of culture treated with supernatants from infected or uninfected macrophages, and binding of tocilizumab to IL-6 receptor and its ability to inhibit STAT3 phosphorylation. [file spectrum.02520-25-s0004.pdf]

A

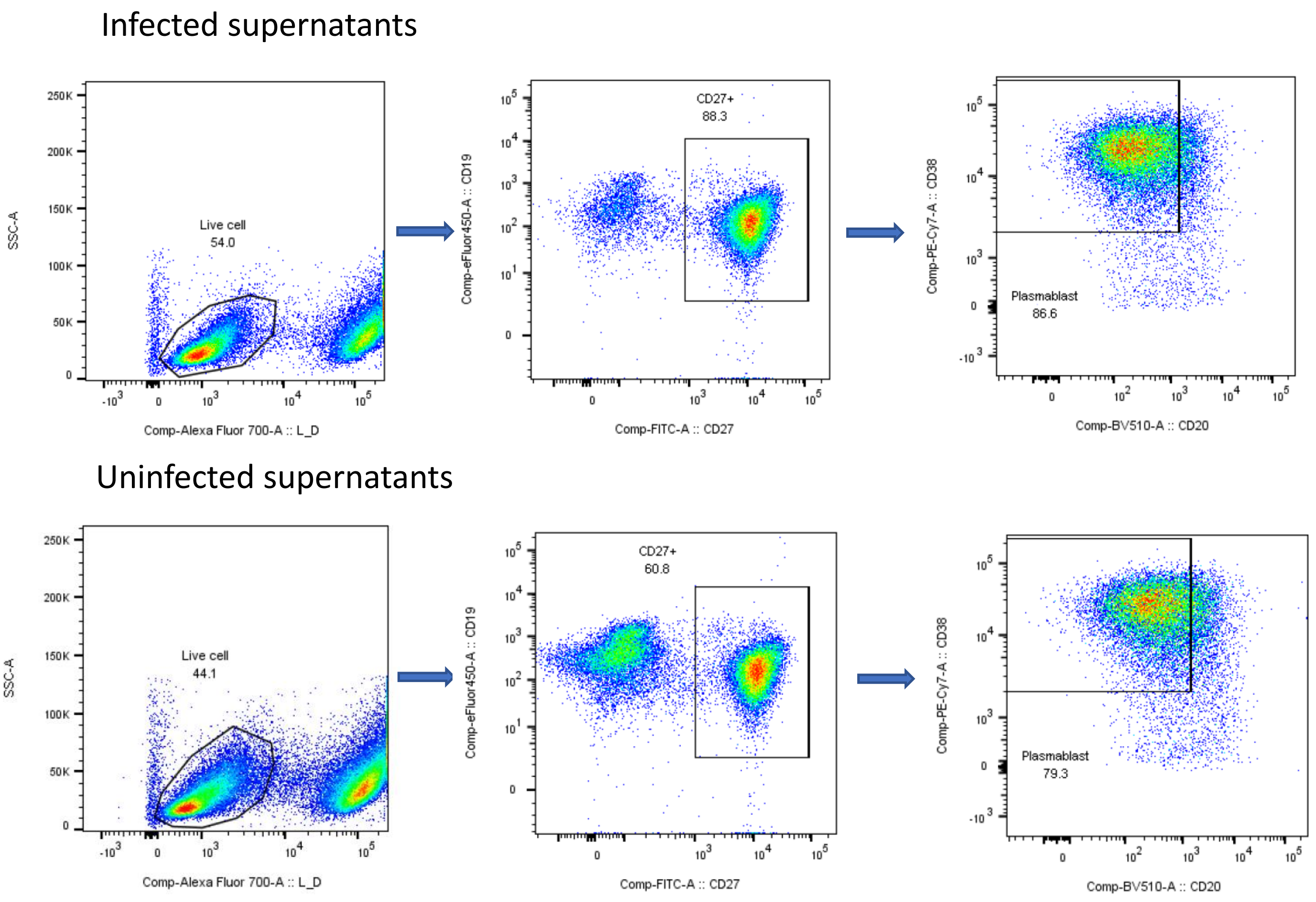

Supplementary Data 6A: Plasmablasts on day 7 of culture treated with supernatants from infected or uninfected macrophages. Supernatants from infected macrophages increased both the percentage of live cells and the frequency of of $CD27^{hi}CD20^{lo}CD38^{hi}$ plasmablasts.

B

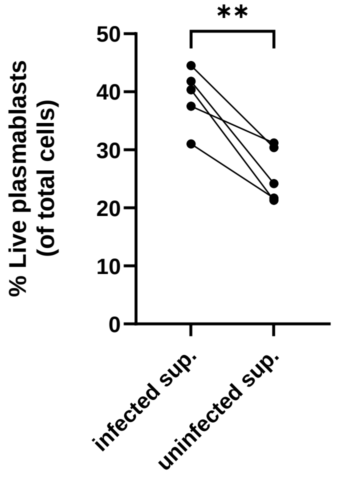

Supplementary Data 6B: Plasmablasts on day 7 of culture treated with supernatants from infected or uninfected macrophages without amikacin treatment.

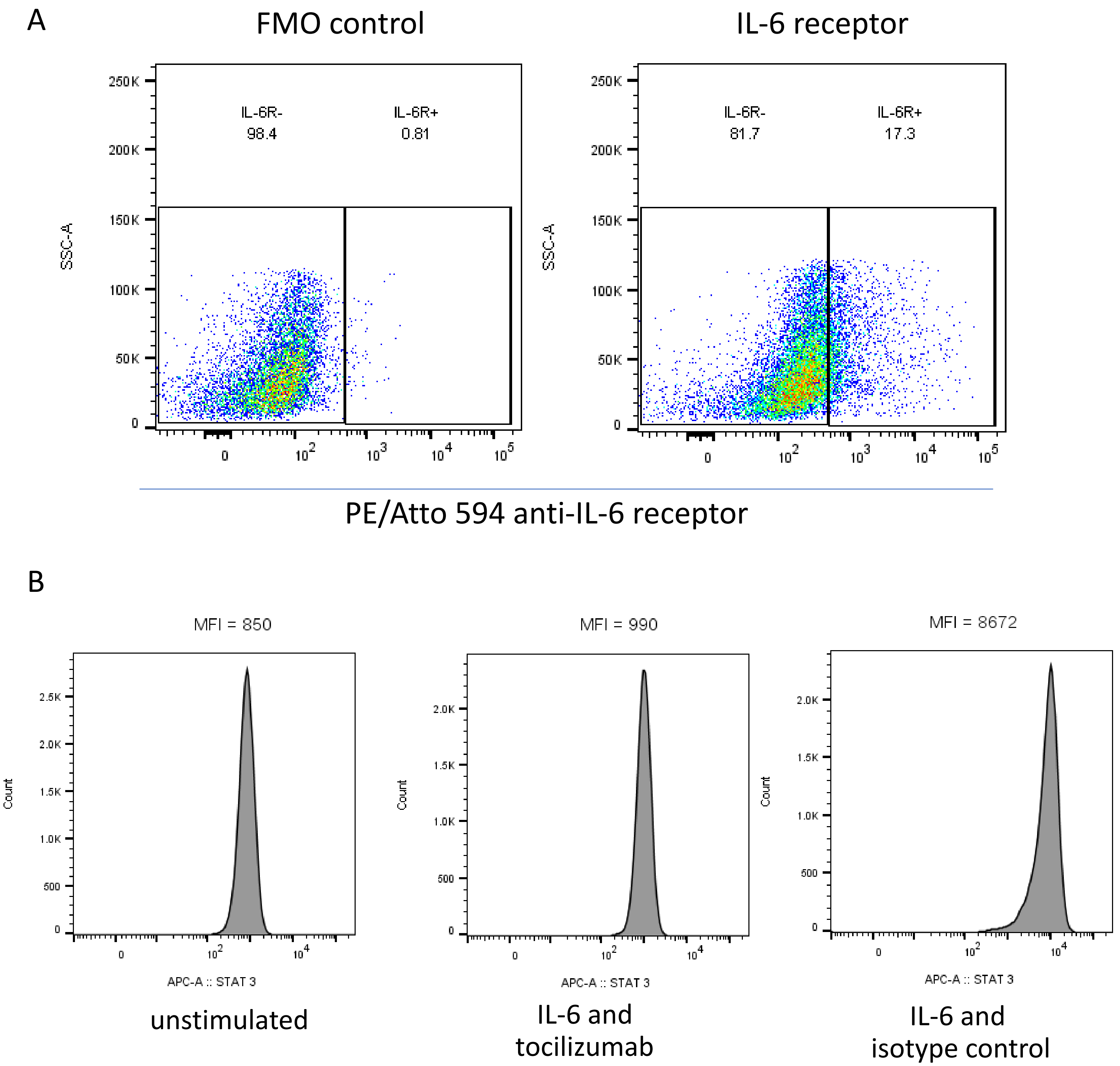

Supplementary Data 7 : Binding of tocilizumab to IL-6 receptor and its ability to inhibit STAT3 phosphorylation

A. Early plasmablasts (day 4) were stained with anti-CD20, anti-CD38, and anti-CD27 antibodies in the presence or absence of PE/Atto 594–labeled tocilizumab. Fluorescence minus one (FMO) control and positive IL-6 receptor staining are shown.

B. THP-1 monocytic cells were left unstimulated or stimulated with IL-6 in the presence of tocilizumab or an isotype control. STAT3 phosphorylation at 20 minutes was assessed by intracellular staining with Alexa Fluor 647–conjugated anti-phospho-STAT3 (Y705).

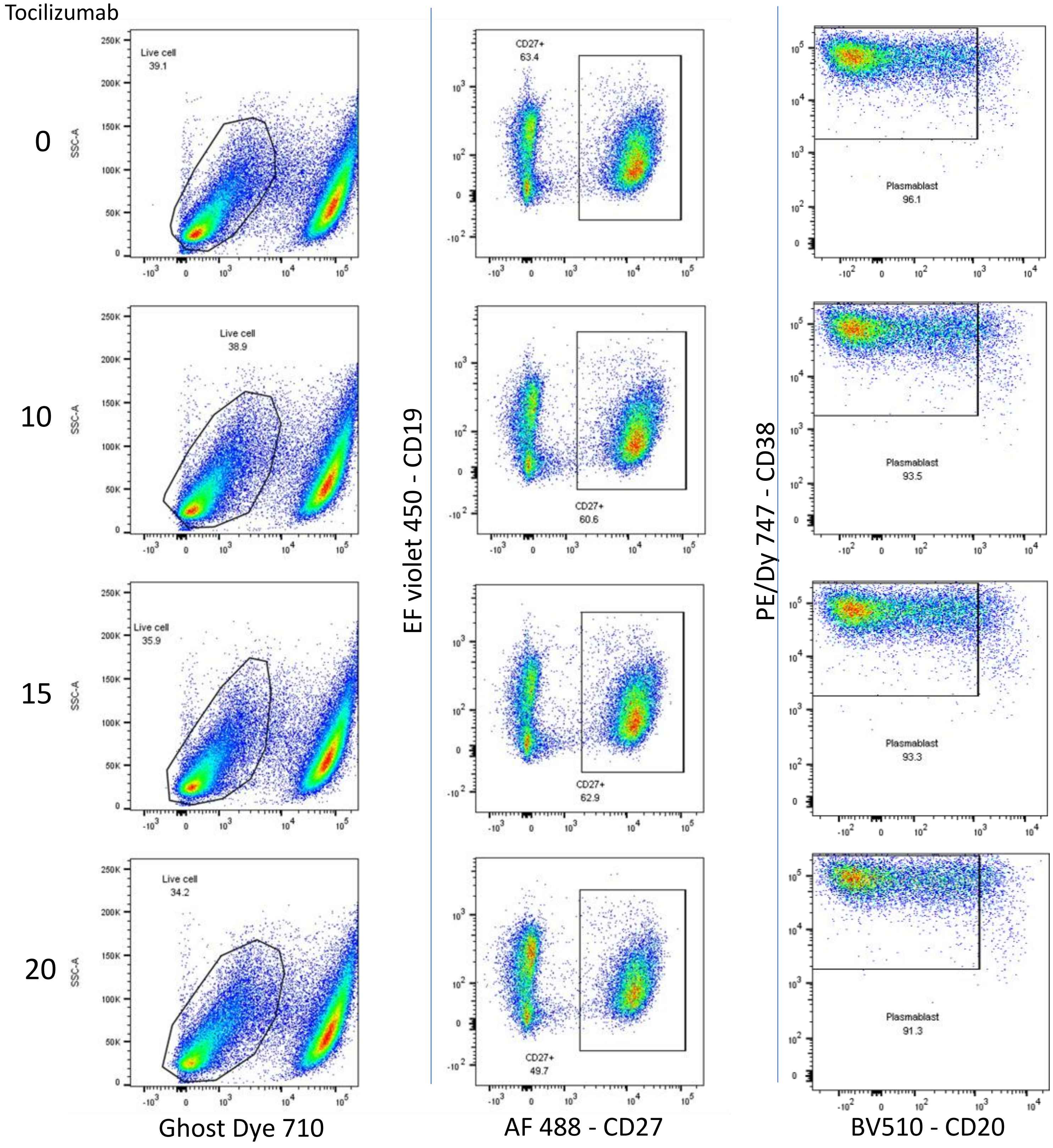

Supp. Data 7C
Day 4 plasmablasts were treated with varying concentrations of tocilizumab, and flow cytometry data from day 7 plasmablasts are shown. Data are representative of two independent experiments.

Tocilizumab (ug/mL)	% Live plasmablasts (of total cells)
0	24.79
10	22.04
15	21.07
20	15.52

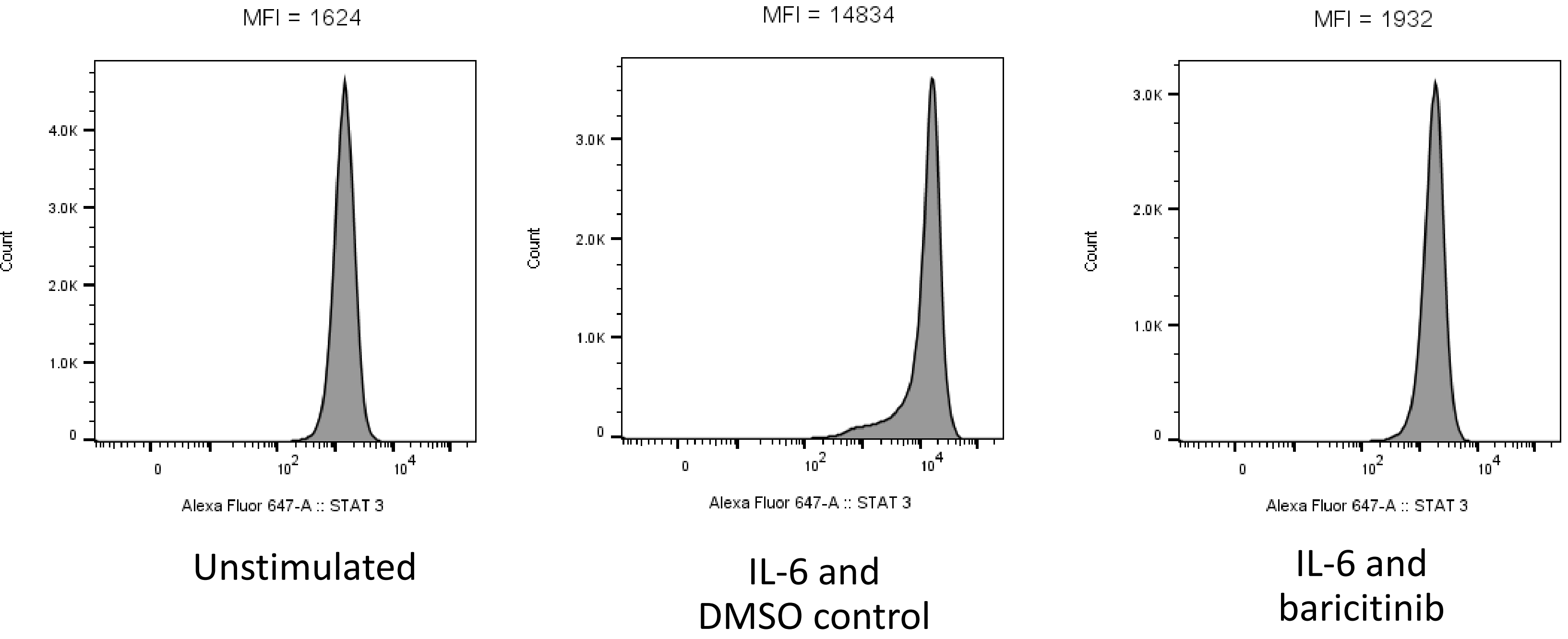

Supplementary Data 7D : Baricitinib inhibits IL-6-mediated STAT3 phosphorylation
THP-1 monocytic cells were left unstimulated or stimulated with IL-6 in the presence of baricitinib (100 nM) or an DMSO control. STAT3 phosphorylation at 20 minutes was assessed by intracellular staining with Alexa Fluor 647–conjugated anti-phospho-STAT3 (Y705).

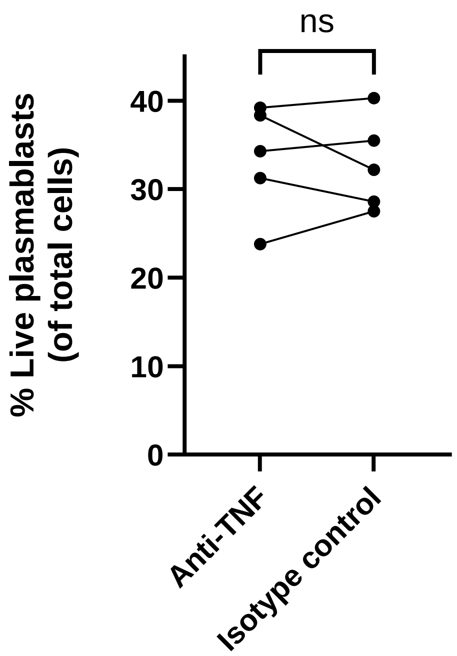

Supplementary Data 7E : Effect of anti-TNF on plasmablast survival and expansion
Plasmablasts on day 4 were treated with either anti-TNF (10 μg/mL) or isotype control in presence of supernatants of infected macrophages. The percentage of live plasmablasts on day 7 was determined by flow cytometry.
