# Supplementary material for: Interleukin-6 from Mycobacterium abscessus-infected macrophages enhances the survival of B cell-derived plasmablasts in vitro
Source: Microbiol Spectr. 2026 Apr 20;14(6):e02520-25. doi: 10.1128/spectrum.02520-25 (PMC13228045; doi:10.1128/spectrum.02520-25)
Supplement: Table S1 — Primer sequences. [file spectrum.02520-25-s0005.pdf]

Supplementary Table 1 Primer sequences

Gene	Sequence (5'→3')
BAFF _F	CGGGACTGAAAATCTTTGAACC
BAFF _R	TGTTTCTTCTGGACCCTGAAC
ARPIL (α, γ isoforms)_F	GGAGCAGAGTTCCGATGC
ARPIL (α, γ isoforms)_R	GACAGAGTGCTGCTTCTTCT
IL-10_F	GGCGCTGTCATCGATTTCT
IL-10_R	TGTAGATGCCTTTCTCTTGGAG
TNF-α_F	CCCAGGCAGTCAGATCATCTTC
TNF-α_R	CAGCTTGAGGGTTTGCTACAAC
IL-6_F	CGGGAACGAAAGAGAAGCTCTA
IL-6_R	GGCGCTTGTGGAGAAGGAG
IL-12/23p40_F	CCTGCAGTTAGGTTCTGATCCA
IL-12/23p40_R	GATTGTTTCAATGAGCATTTAGCATC
IL-23/p19_F	GTGGGACGCGATCTAAGAG
IL-23/p19_R	CCTTTGCAAGCAGAACTGAC
GADPH_F	ACAGCCTCAAGATCATCAGCA
GADPH_R	GATGGCATGGACTGTGGTCA
